# Magnitude and trends of inequalities in antenatal care and delivery under skilled care among different socio-demographic groups in Ghana from 1988 – 2008

**DOI:** 10.1186/1471-2393-14-295

**Published:** 2014-08-29

**Authors:** Benedict O Asamoah, Anette Agardh, Karen Odberg Pettersson, Per-Olof Östergren

**Affiliations:** Department of Clinical Sciences, Social Medicine and Global Health, Lund University, Malmö, Sweden

## Abstract

**Background:**

Improving maternal and reproductive health still remains a major challenge in most low-income countries especially in sub-Saharan Africa. The growing inequality in access to maternal health interventions is an issue of great concern. In Ghana, inadequate attention has been given to the inequality gap that exists amongst women when accessing antenatal care during pregnancy and skilled attendance at birth. This study therefore aimed at investigating the magnitude and trends in income-, education-, residence-, and parity-related inequalities in access to antenatal care and skilled attendance at birth.

**Methods:**

A database was constructed using data from the Ghana Demographic and Health Surveys (DHS) 1988, 1993, 1998, 2003, and 2008. The surveys employed standard DHS questionnaires and techniques for data collection. We applied regression-based Total Attributable Fraction (TAF) as an index for measuring socioeconomic inequalities in antenatal care and skilled birth attendance utilization.

**Results:**

The rural–urban gap and education-related inequalities in the utilization of antenatal care and skilled birth attendants seem to be closing over time, while income- and parity-related inequalities in the use of antenatal care are on a sharp rise. Income inequality regarding the utilization of skilled birth attendance was rather low and stable from 1988 to 1998, increased sharply to a peak between 1998 and 2003, and then leveled-off after 2003.

**Conclusions:**

The increased income-related inequalities seen in the use of antenatal care and skilled birth attendance should be addressed through appropriate strategies. Intensifying community-based health education through media and door-to-door campaigns could further reduce the mentioned education- and parity-related inequalities. Women should be highly motivated and incentivized to attend school up to secondary level or higher. Education on the use of maternal health services should be integrated into basic schools so that women at the lowest level would be inoculated with the appropriate health messages.

## Background

Improving maternal and reproductive health still remains a major challenge in most low-income countries, especially in sub-Saharan Africa [1–3]. More than a decade ago the global community committed itself to 8 Millennium Development Goals (MDGs). MDG 5 focuses on improving maternal health and has two targets: 5a, to reduce maternal mortality ratio by 75% between 1990 and 2015, and 5b, to achieve universal access to reproductive health. Following the millennium declaration, there have been some improvements in the uptake of maternal health interventions such as antenatal care (ANC), skilled birth attendance (SBA), and facility-based delivery [4, 5] but hardly any in resource poor countries that bear the highest burden of maternal mortality [3]. Moreover, within these countries, the most vulnerable women have the worst maternal health outcomes [6, 7].

SBA is one of the main proxy indicators globally adopted to monitor progress in MDG 5. Several studies have presented evidence of an association between SBA utilization during childbirth and reduction in maternal mortality [8–11]. The definition of a SBA has evolved over the years. In 2004, the WHO, International Confederation of Midwives (ICM) and the International Federation of Gynecology and Obstetrics (FIGO) refined the definition of a SBA as “an accredited health professional – such as a midwife, doctor or nurse – who has been trained to proficiency in the skills needed to manage normal (uncomplicated) pregnancies, childbirth and the immediate postnatal period, and in the identification, management and referral of complications in women and newborns” [12]. The above definition developed due to the realisation that diverse groups of health care professionals with country-specific titles could provide the skills and competencies expected of a SBA [13–15].

The use of SBA during childbirth is almost universal in high income countries, but lags behind in most low-income settings in sub-Saharan Africa [16], despite it being an indisputable benefit [17, 18]. The unavailability of maternal health care services generally accounts for the low use of skilled care during childbirth [19]. However, even in settings where these services are available, women with low socio-economic status, minimal education, and with residence in rural areas fail to access them. Reasons for this include high direct and indirect costs of healthcare, lack of transportation, long distances to health facilities, inadequate information about services provided, and negative past experiences with health care providers [19–21]. Socio-cultural vulnerabilities also play a role in inhibiting women’s use of skilled care at birth. For example, some women place a high value on home birth and fear they will lose social status, confidentiality, and control over the birth process with assisted delivery [21]. In some instances, the decision to deliver at a facility is not up to the woman but is made by husbands, mother-in-laws, community heads, soothsayers or traditional healers [22, 23]. Many women prefer and seek to give birth under conditions where they feel safe, protected and secure [24–26].

ANC is one of the factors that tend to promote delivery with a skilled health professional [27, 28]. In most low-income countries, women may attend at least one ANC visit during pregnancy [29], but fail to adhere [30] to the WHO recommendation of at least four ANC visits for a given pregnancy [31, 32].

The growing inequality in access to health care and in health outcomes is an issue of concern as countries strive to improve maternal health. SBA utilization is one of the indicators that have been selected by the WHO Equity Analysis Group because of its relevance to health services and health system strength [33]. A recent study conducted in 54 low-income countries in which 12 maternal, newborn, and child health interventions were analyzed, found SBA coverage to be the least equitable one, followed by completing four or more ANC visits [30]. The same study found that countries with similar levels of overall SBA coverage often had very different results in regard to the distribution of these interventions by wealth quintiles [30].

Utilization of ANC and SBA is very low in Ghana. The proportion of births attended by SBAs was 57% in 2008 [34], far below the UN minimum target of 80% in 2005, 85% in 2010, and 90% in 2015. Moreover, differences exist in uptake and quality of these services within the country [35, 36]. Also, more attention has been focused on attaining set international targets in ANC and SBA coverage, but inadequate attention has been given to the inequality gap that exists in women who access the services.

Inequalities in health vary with time, place and by specific health indicators [30]. Whereas aggregate measures of health inequality could be used in certain instances, such as for global monitoring, they are less useful at the country level in guiding decisions on policy and interventions than are single indicators [30]. The magnitude of and trends in inequality regarding SBA coverage and adequate ANC utilization in Ghana has not yet been studied. With the socio-demographic disparities that exist in Ghana, especially in education, income, and rural/urban residence status, it is essential to investigate the trend in social disparities and how these have translated into inequalities in accessing adequate ANC and SBA. This study therefore aimed at investigating the magnitude of and trends in income-, education-, residence-, and parity-related inequalities in accessing adequate ANC and skilled care at birth.

## Methods

### Data collection

A database was constructed using data from the Ghana Demographic and Health Survey of 1988 [37], 1993 [38], 1998 [39], 2003 [40], and 2008 [34], which employed standard DHS questionnaires and techniques for data collection [34, 37–40]. All participants were interviewed using the Women’s Questionnaire. Eligible women were defined as women aged 15 to 49 who stayed in a selected household the night before the interview, whether or not they were usual residents in the household. The Women’s Questionnaire was used to collect information on the following topics: respondent’s background characteristics; reproductive history; contraceptive knowledge and use; antenatal, delivery and postnatal care; infant feeding practices; child immunization and health; marriage; fertility preferences and attitudes about family planning; husband’s background characteristics; women’s work; knowledge of STDs including HIV/AIDS; and anthropometric measurements of children and mothers. The actual study analysed antenatal and delivery experiences of women with at least one previous birth experience in the last 3 to 5 years prior to the surveys. Response rates for the Women’s Questionnaire were between 96% and 99% over the study years. Response rates on questions related to the use of ANC varied from 95.6 to 98.7% and that of SBA from 99.6 to 99.8%.

### Definition of variables

#### Dependent variables

Two outcome variables were used to assess trends in pregnancy or birth experiences, namely, *number of antenatal care visits and skilled attendance at birth*. The maternity history contained up to six entries relating to births in the last three to five years, depending on the year of survey. For women with multiple birth experiences, the last birth experience was analysed in this study.

1. *Antenatal care visits*. ANC visits were accessed using two variables: a) no ANC visit or at least one visit, and b) less than four ANC visits or at least four visits.
2. *Skilled attendance at birth.* This variable was generated from response to the question that assessed “the type of person that assisted in the delivery of the child”. Responses were dichotomized as a) women who had skilled attendance at birth from a doctor, nurse, or midwife), and b) those who had no skilled attendance at birth. Auxiliary health staff and health assistants included in the 2008 survey were not considered as SBAs.

#### Independent variables

The independent variables used in this study were as follows:

1. *Maternal age.* This variable was categorized into three age groups (below 25, 25–34 and 35 or above)
2. *Educational level*. This was classified into four categories:
  - Never attended school (women who confirmed having no formal education)
  - Basic education (women with some level of formal education not exceeding 9 years, including those with primary, middle school, or lower secondary school education)
  - Senior high school (women with 12 years of formal education or whose education ended at the upper secondary school level). Those who completed 12 years of basic and secondary education and had some extra years of education, but did not complete tertiary level education, were also included in this category.
  - Tertiary or higher education (women who completed at least 15 years of formal education, including those with college, polytechnic, or university level studies).

Educational level was re-categorized into three categories: no education, basic education, and senior high school or higher (secondary +)

3. *Residence*. Residence was coded as either urban or rural
4. *Current marital status*. Current marital status was classified in two categories:
  - Single (women who had never married, were separated, divorced, or widowed at the time of the interview)
  - Married (women who were married or living with a partner at the time of the interview)
5. *Income level*. Income level was calculated based on the yearly earnings of the respondents. This variable was originally categorized into five quintiles (poorest, poorer, middle, richer, richest) according to the Ghana Demographic and Health Survey. Income quintiles were later ranked into three groups: low income (poorest and poorer), average income (middle) and high income (richer and richest) using the fractional rank function in SPSS.
6. *Parity*. This variable was coded from a question that assessed the number of children a woman had ever given birth to. Responses were grouped as nulliparous (zero births prior to the current pregnancy), para 1 to 3 (1 to 3 births) and para 4 + (4 or more births).

### Statistical methods and analysis

#### Measures of inequality

In this study, we applied regression-based Total Attributable Fraction (TAF) [41], which we consider a robust index for measuring inequalities in health. The IBM software SPSS Statistics 20 and Microsoft Excel were used for analysis. The independent variables included in the logistic regression model were selected based on the aim of our study, and the importance of the covariates to our chosen outcomes based on a review of previous literature. Our main exposure variables (education, residence, income, and parity) were chosen so as to produce a model that is stable enough and has relevant policy implications. Therefore, variables with unstable characteristics in the Ghanaian context such as religion and ethnicity were not included. Also, other covariates with the mentioned characteristics were tested in a univariate analysis to see if they were at least moderately associated with the outcome.

##### Total Attributable Fraction (TAF)

TAF represents the proportion of the outcome that would not exist if all women had had the same prevalence as those with the highest socioeconomic status, the assumption being that there is a causal pathway between socioeconomic status and the outcome variable. The attributable fraction was calculated using the formula AF = (OR - 1)/OR, where OR is the adjusted odds ratio by logistic regression analysis. TAF was calculated as follows: **TAF = ∑ (sTAF) = ∑AF**_i_*P_**i,**_ where AF_i_ is the attributable fraction for the outcome variable for a specific stratum, and P_i_ represents the proportion of all cases that fall within this stratum. The Product of AF_i_ and P_i_ represents the stratum-specific Total Attributable Fraction (sTAF), and **∑ (sTAF)** indicates the summation of all the strata-specific calculations, referred to as the overall TAF. For those with the highest level of education, the AF and sTAF are by definition zero. DHS sampling design uses clustered sampling, which includes both over-sampling and under-sampling. In regions with small populations, over-sampling is done to ensure that there is a large enough sample to be representative, while under-sampling is done in regions with large populations as a means of saving cost. Initial sample weights are produced using the sample selection probabilities of each household, and response rates for the households and the individuals. The initial weights are then standardized by dividing each weight by the average of the initial weights (equal to the sum of the initial weight divided by the sum of the number of cases). This ensures that the sum of the standardized weights equals the sum of the cases over the entire sample. Therefore, all analysis were conducted with sample-weighted data to correct for the over-sampling, the under-sampling, and the different response rates to the survey in different regions. Further adjustments were made for the clustering at the individual and household levels.

## Results

Table 1 shows the characteristics of the study sample. The number of respondents totaled 2716 in 1988, 1980 in 1993, 2376 in 1998, 2777 in 2003, and 2147 in 2008. The majority of participants were within 25–34 years, had basic education, resided in rural areas and were married. For one fifth of the respondents, it was their first birth experience while the majority had more than one birth experience (para 1 to 3: 44–50% and para 4 +: 28–36% between 1988 and 2008). From 1993 to 2008, the proportion of women with at least one ANC visit increased from 87.1% to 96.1%, those with at least four visits increased from 60.1% to 78.7%, while the proportion of births attended by skilled health professionals increased from 43.8% to 54.0% within the same period.

**Table 1:** **Socio-demographic characteristics, antenatal care, delivery under skilled care and valid percent (%) among Ghanaian women 15–49 years with birth history from 1988 to 2008 presented in 5 years intervals**

	Year				
	1988	1993	1998	2003	2008
**Age**					
<25	763 (28.1)	600 (30.3)	617 (26.0)	649 (23.4)	518 (24.1)
25-34	1249 (46.0)	957 (48.3)	1055 (44.4)	1288 (46.4)	985 (45.9)
35+	704 (25.9)	423 (21.4)	704 (29.6)	840 (30.2)	644 (30.0)
Total	2716 (100)	1980 (100)	2376 (100)	2777 (100)	2147 (100)
**Residence**					
Urban	771 (28.4)	567 (28.6)	555 (23.4)	817 (29.4)	763 (35.5)
Rural	1945 (71.6)	1413 (71.4)	1821 (76.6)	1960 (70.6)	1384
Total	2716 (100)	1980 (100)	2376 (100)	2777 (100)	(64.5) 2147 (100)
**Highest educational level**					
No education	1197 (44.1)	795 (40.2)	1062 (44.7)	1272 (45.8)	774 (36.1)
Basic education	1376 (50.7)	1072 (54.1)	1177 (49.5)	1361 (49.0)	1180 (55.0)
Secondary	126 (4.6)	98 (4.9)	113 (4.8)	113 (4.1)	143 (6.7)
Higher	17 (0.6)	15 (0.8)	24 (1.0)	31 (1.1)	48 (2.2)
Total	2716 (100)	1980 (100)	2376 (100)	2777 (100)	2145 (100)
Missing	0	0	0	0	2
**Marital status**					
Married	2396 (88.2)	1807 (91.3)	2109 (88.8)	2509 (90.3)	1907 (88.8)
Single	319 (11.7)	173 (8.7)	267 (11.2)	268 (9.7)	240 (11.2)
Total	2715 (100)	1980 (100)	2376 (100)	2777 (100)	2147 (100)
Missing	1	0	0	0	0
**Income level**					
Low income	595 (40.7)	No data	772 (44.0)	1482 (53.4)	1107 (51.6)
Average income	320 (21.9)		364 (20.8)	503 (18.1)	375 (17.5)
High income	547 (37.4)		618 (35.2)	792 (28.5)	665 (31.0)
Total	1462 (100)		1754 (100)	2777 (100)	2147 (100)
Missing	1254		622	0	0
**Parity**					
Nulliparous	515 (19.0)	400 (20.2)	512 (21.5)	578 (20.8))	457 (21.3)
Para 1-3	1206 (44.4)	7996 (50.3)	1107 (46.6)	1288 (46.4)	1089 (50.7)
Para 4+	995 (36.6)	584 (29.5)	757 (31.9)	911 (32.8)	601 (28.0)
Total	2716 (100)	1980 (100)	2376 (100)	2777 (100)	2147 (100)
**Less than one antenatal visit**					
No	No data	1702 (87.1)	2070 (88.8)	2445 (92.1)	1995 (96.1)
Yes		253 (12.9)	261 (11.2)	211 (7.9)	82 (3.9)
Total		1955 (100)	2331 (100)	2656 (100)	2077 (100)
Missing		25	45	121	70
**Less than four antenatal visits**					
No	No data	1174 (60.1)	1502 (64.4)	1881 (70.6)	1644 (78.7)
Yes		781 (39.9)	829 (35.6)	782 (29.4)	444 (21.3)
Total		1955 (100)	2331 (100)	2663 (100)	2088 (100)
Missing		25	45	114	59
**Lack of skilled attendant at birth**					
No	1120 (41.4)	866 (43.8)	989 (41.7)	1238 (44.8)	1157 (54.0)
Yes	1583 (58.6)	1112 (56.2)	1382 (58.3)	1527 (55.2)	987 (46.0)
Total	2703 (100)	1978 (100)	2371 (100)	2765 (100)	2144 (100)
Missing	13	2	5	12	3

Table 2 presents the prevalence of women with less than four ANC visits and those who had no skilled care at birth according to their socioeconomic characteristics, from 1988 to 2008. There was a general reduction in the prevalence of women who had less than four ANC visits during their last pregnancy, and those who had no skilled attendance at birth within the different socioeconomic groups. From 1993 to 2008, the utilization of ANC increased dramatically among women residing in rural areas (prevalence difference, 21.5%), those with no education (prevalence difference, 25.7%), and those who had only basic level education (prevalence difference, 13.9%), compared to those with urban residency and secondary or higher education. Within the income levels, increased utilization of ANC was mostly attributed to high-income women. The prevalence of four or more antenatal care visits increased comparably across all parity groups (prevalence differences between 1993 and 2008: nulliparous 17.6%, para 1 to 3 19.7%, para 4 + 17.2%). The increased utilization of ANC seen in the socioeconomic groups did not reflect in the use of SBA within those groups. The utilization of SBA increased mainly in women with urban residence, high education, high income, and low parity.

**Table 2:** **Prevalence of less than four antenatal visits and lack of skilled attendant at birth according to socio-demographic characteristics among Ghanaian women 15–49 years from 1988 to 2008 presented in 5 years intervals**

	Less than four antenatal visits				Lack of skilled attendant at birth				
	1993	1998	2003	2008	1988	1993	1998	2003	2008
	n (%)	n (%)	n (%)	n (%)	n (%)	n (%)	n (%)	n (%)	n (%)
**Residence**									
Urban	94 (16.9)	97 (18.2)	91 (11.8)	73 (9.8)	219 (28.6)	105 (18.6)	128 (23.1)	160 (19.6)	138 (18.1)
Rural	687 (49.1)	732 (40.7)	691 (36.5)	371 (27.6)	1364 (70.4)	1007 (71.3)	1254 (69.0)	1367 (70.1)	849 (61.5)
Total	781	829	782	444	1583	1112	1382	1527	987
**Highest educational level**									
No education	433 (55.4)	484 (46.4)	475 (39.3)	220 (29.7)	880 (73.9)	608 (76.6)	825 (77.8)	920 (72.7)	517 (67.0)
Basic education	341 (32.1)	338 (29.3)	300 (22.8)	211 (18.2)	675 (49.3)	487 (45.5)	539 (45.9)	593 (43.7)	448 (38.0)
Secondary +	7 (6.2)	7 (5.2)	7 (5.0)	13 (7.0)	28 (19.7)	17 (15)	18 (13.1)	14 (9.7)	22 (11.5)
Total	781	829	782	444	1583	1112	1382	1527	987
**Income level**									
Low income	No data	304 (39.9)	551 (38.6)	326 (30.5)	387 (65.5)	No data	532 (69.2)	1120 (76.0)	753 (68.2)
Average income		112 (31.0)	156 (32.0)	77 (20.8)	166 (52.2)		188 (51.6)	270 (53.8)	135 (36.0)
High income		148 (24.7)	75 (10.0)	41 (6.3)	235 (43.1)		261 (42.3)	137 (17.4)	99 (14.9)
Total		564	782	444	788		981	1527	987
**Parity**									
Nulliparous	137 (34.6)	150 (30.1)	133 (23.6)	75 (17.0)	245 (47.9)	174 (43.5)	224 (43.8)	245 (42.4)	152 (33.3)
Para 1-3	386 (39.2)	365 (33.5)	332 (27.3)	207 (19.5)	714 (59.5)	558 (56.1)	620 (56.1)	690 (53.9)	475 (43.7)
Para 4+	258 (44.9)	314 (42.3)	317 (35.9)	162 (27.7)	624 (63.2)	380 (65.1)	538 (71.3)	592 (65.3)	360 (60.1)
Total	781	829	782	444	1583	1112	1382	1527	987

Table 3 gives the adjusted odds ratios and 95% confidence intervals for having less than four antenatal visits and lack of skilled attendant at birth, from 1988 to 2008. A significant positive association was seen between women who attended less than four antenatal care visits and those who had rural residence, low education, low income, and high parity through out the study period. There was a declining trend in the odds of having less than four antenatal visits for women of rural background (OR _adjusted_ 3.5 in 1993, 2.3 in 1998, 2.4 in 2003 and 1.5 in 2008), no education (OR _adjusted_ 10.4 in 1993, 10.7 in 1998, 4.4 in 2003, 2.2 in 2008), and basic level education (OR _adjusted_ 4.4 in 1993, 5.5 in 1998, 2.6 in 2003, 1.5 in 2008). The reverse occurred in low income and high parity women (OR _adjusted (Para 1–3)_ 1.1 in 1993, 1.5 in 1998, 1.3 in 2003, 1.8 in 2008, and OR _adjusted (Para 4+)_ 1.3 in 1993, 2.0 in 1998, 2.3 in 2003, 3.5 in 2008) within the same period. We also observed a significant positive association between lack of skilled attendant at birth and having rural residence status (OR _adjusted_ 4.4 in 1988, 8.3 in 1993, 4.3 in 1998, 3.0 in 2003, 2.4 in 2008), no education (OR _adjusted_ 5.1 in 1988, 6.7 in 1993, 6.7 in 1998, 6.3 in 2003, 3.8 in 2008), up to basic level education (OR _adjusted_ 2.2 in 1988, 2.2 in 1993, 2.6 in 1998, 3.2 in 2003, 1.9 in 2008), low income, and high parity from 1988 to 2008.

**Table 3:** **Adjusted odds ratios with 95% Confidence intervals showing the association between education, income, residence and parity, and less than four antenatal visits and lack of skilled attendant among Ghanaian women 15–49 years from 1988 to 2008 presented in years intervals**

Variables	Less than four antenatal visits				Lack of skilled attendant at birth				
	Adjusted* OR 95% CI				Adjusted* OR (95% CI)				
	1993	1998	2003	2008	1988	1993	1998	2003	2008
**Residence**									
Urban	Ref	Ref	Ref	Ref	Ref	Ref	Ref	Ref	Ref
Rural	3.5 (2.7-4.5)	2.3 (1.7-3.1)	2.4 (1.7-3.2)	1.5 (1.1-2.1)	4.4 (3.4-5.8)	8.3 (6.4-10.6)	4.3 (3.3-5.6)	3.0 (2.4-3.9)	2.4 (1.9-3.2)
**Highest educational level**									
No education	10.4 (4.7-23.2)	10.7 (4.4-25.7)	4.4 (2.0-9.4)	2.2 (1.2-4.4)	5.1 (2.9-9.0)	6.7 (3.6-12.2)	6.7 (3.7-11.8)	6.3 (3.4-11.4)	3.8 (2.3-6.4)
Basic education	4.4 (2.0-9.7)	5.5 (2.3-13.0)	2.6 (1.2-5.5)	1.5 (0.8-2.9)	2.2 (1.3-3.8)	2.2 (1.3-4.0)	2.6 (1.5-4.6)	3.2 (1.8-5.8)	1.9 (1.1-3.1)
Secondary +	Ref	Ref	Ref	Ref	Ref	Ref	Ref	Ref	Ref
**Income level**	No data					No data			
Low income		1.5 (1.2-1.9)	2.3 (1.6-3.2)	3.5 (2.3-5.3)	1.7 (1.3-2.3)		1.6 (1.2-2.0)	4.3 (3.3-5.7)	4.5 (3.3-5.9)
Average income		1.2 (0.9-1.6)	2.3 (1.7-3.2)	2.8 (1.9-4.2)	1.3 (0.9-1.7)		1.1 (0.9-1.5)	2.7 (2.1-3.6)	2.1 (1.6-2.9)
High income		Ref	Ref	Ref	Ref		Ref	Ref	Ref
**Parity**									
Nulliparous	Ref	Ref	Ref	Ref	Ref	Ref	Ref	Ref	Ref
Para 1-3	1.1 (0.8-1.6)	1.5 (1.1-2.1)	1.3 (1.0-1.8)	1.8 (1.3-2.6)	1.6 (1.1-2.3)	1.8 (1.3-2.5)	1.9 (1.3-2.6)	1.8 (1.3-2.4)	1.5 (1.1-2.1)
Para 4+	1.3 (0.9-1.9)	2.0 (1.3-3.1)	2.3 (1.6-3.4)	3.5 (2.2-5.7)	1.6 (1.0-2.7)	2.0 (1.3-3.1)	3.5 (2.3-5.5)	2.6 (1.8-3.8)	2.2 (1.4-3.3)

*Mutually adjusted for one another, age, marital status, and the clustering at individual and household levels.

**Figure 1:**
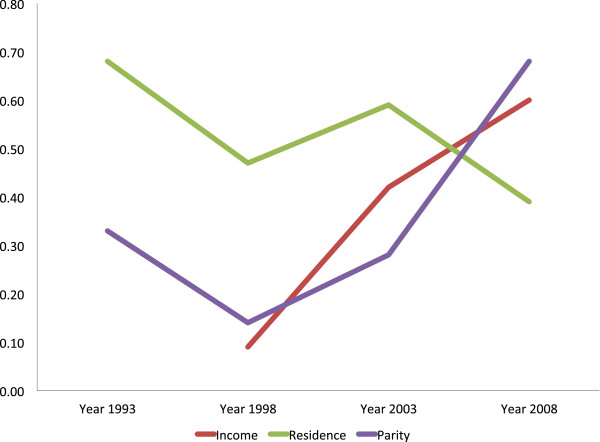
**Inequality trends in attending at least one antenatal care visit in Ghana, 1993 to 2008 [TAF = Total Attributable Fraction].**

**Figure 2:**
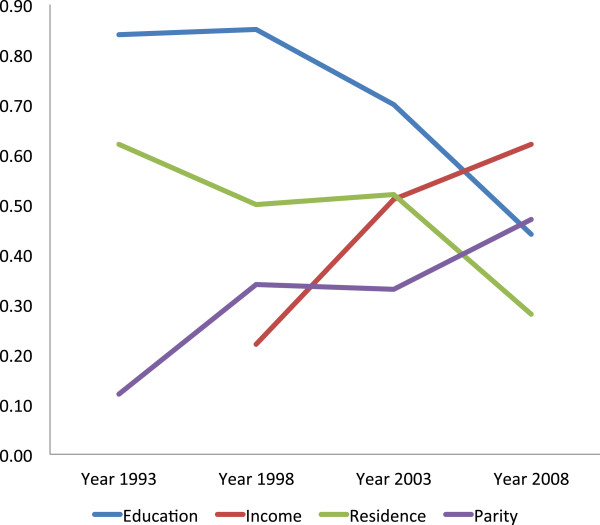
**Inequality trends in attending at least four antenatal care visits in Ghana, 1993 to 2008 [TAF = Total Attributable Fraction].**

**Figure 3:**
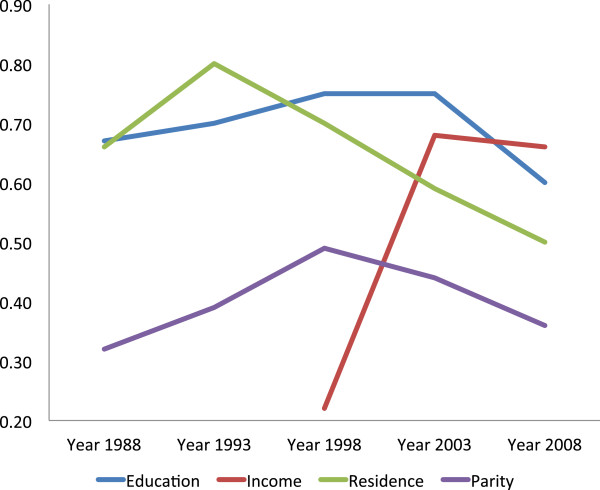
**Inequality trends in utilization of skilled birth attendance in Ghana, 1988 to 2008 [TAF = Total Attributable Fraction].**

**Figure 4:**
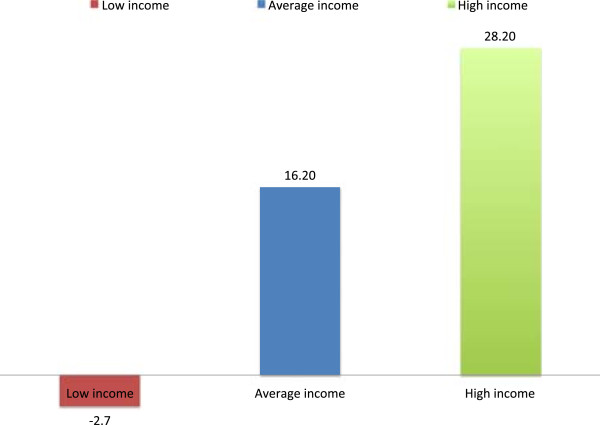
**Absolute change (%) in the prevalence of SBA utilization in Ghana from 1988 to 2008 by income levels.**

Residence- and education-related inequality (measured by means of TAF) in utilization of ANC (at least one and at least four visits) declined remarkably from 1993 to 2008, while income- and parity-related inequalities in the same outcome increased (Table 4, Figures 1 and 2). The residence-related inequality in attending at least one ANC visit was 0.68 in 1993, 0.47 in 1998, 0.59 in 2003 and 0.39 in 2008, whereas that of the recommended minimum, of four visits, declined from 0.62 in 1993 to 0.50 in 1998, 0.52 in 2003, and 0.28 in 2008. TAF for education-related inequality in having at least four ANC visits was 0.84 in 1993, 0.85 in 1998, 0.70 in 2003, and 0.44 in 2008. However, TAF regarding income-related inequality in attending at least four ANC visits increased from 0.22 in 1998 to 0.51 in 2003, and 0.62 in 2008, similar to parity-related inequality in the same outcome (TAF = 0.12 in 1993, 0.34 in 1998, 0.33 in 2003, and 0.47 in 2008). The increased utilization of ANC by women with low level of education and those who had rural residence status did not directly translate into utilization of SBA as would be expected (Residence-related TAF_1988–2008_: were 0.66 in 1988, 0.80 in 1993. 0.70 in 1998, 0.59 in 2003, 0.50 in 2008, and education–related TAF_1988–2008_: 0.67 in 1988, 0.70 in 1993, 0.75 in 1998, 0.75 in 2003, and 0.60 in 2008). Parity-related inequality in the utilization of SBA showed an upward trend from 1988 to 1998, where it peaked, and began to decline from 1998 to 2008 (Parity–related TAF: 0.32 in 1988, 0.39 in 1993, 0.49 in 1998, 0.44 in 2003, and 0.36 in 2008) (Table 4, Figures 3 and 4). Income-related inequality in the utilization of SBA remained steady from 1988 to 1998, increased sharply from 1998 to 2003, and then remained stable between 2003 and 2008 (Income–related TAF: 0.24 in 1988, 0.22 in 1998, 0.68 in 2003, and 0.66 in 2008) (Table 4, Figures 3 and 4). The sharp increase in income disparities recorded between 1998 and 2003 was attributable to women in both the low income (sTAF_1998, 2003_ = 0.20, 0.57 respectively), and average income (sTAF_1998, 2003_ = 0.02, 0.11 respectively) strata, whereas, from 2003 to 2008, only low income women recorded an increase, though slightly (sTAF_2008_ = 0.59 for low income women and sTAF_2008_ = 0.07 for average income women).

**Table 4:** **Logistic regression-based Attributable Fraction (AF), Stratum-specific Total Attributable Fraction (sTAF) and overall Total Attributable Fraction (TAF) of not attending any antenatal visit, insufficient antenatal visit (<4 visits) and lack of skilled attendant at birth in each stratum from 1988 to 2008 presented in 5 years intervals**

No antenatal care visit					
	1988	1993	1998	2003	2008
	AF (sTAF)	AF (sTAF)	AF (sTAF)	AF (sTAF)	AF (sTAF)
**Residence** ^*^					
Urban		Ref	Ref	Ref	Ref
Rural		0.73 (0.68)	0.52 (0.47)	0.62 (0.59)	0.44 (0.39)
TAF		0.68	0.47	0.59	0.39
p-value		0.000	0.035	0.001	0.211
**Highest educational level** ^*^					
No education		0.94 (0.68)	0.92 (0.70)	-	0.41 (0.21)
Basic education		0.76 (0.20)	0.77 (0.18)		0.16 (0.08)
Secondary+		Ref	Ref		Ref
TAF		0.88	0.88		0.29
p-value		0.000	0.000		0.439
**Income level** ^*^					
Low income		No data	0.17 (0.09)	0.47 (0.39)	0.68 (0.54)
Average income			-	0.23 (0.03)	0.47 (0.06)
High income			Ref	Ref	Ref
TAF			0.09	0.42	0.60
p-value			0.455	0.095	0.056
Parity*					
Nulliparous		Ref	Ref	Ref	Ref
Para 1-3		0.38 (0.19)	0.16 (0.07)	0.09 (0.04)	0.67 (0.32)
Para 4+		0.38 (0.14)	0.16 (0.07)	0.50 (0.24)	0.82 (0.36)
TAF		0.33	0.14	0.28	0.68
p-value		0.151	0.832	0.026	0.007
**Less than four antenatal visits**					
	**1988**	**1993**	**1998**	**2003**	**2008**
	**AF (sTAF)**	**AF (sTAF)**	**AF (sTAF)**	**AF (sTAF)**	**AF (sTAF)**
**Residence** ^*^					
Urban		Ref	Ref	Ref	Ref
Rural		0.71 (0.62)	0.57 (0.50)	0.58 (0.52)	0.33 (0.28)
TAF		0.62	0.50	0.52	0.28
p-value		0.000	0.000	0.000	0.013
**Highest educational level** ^*^					
No education		0.90 (0.50)	0.90 (0.52)	0.77 (0.47)	0.55 (0.28)
Basic education		0.77 (0.34)	0.82 (0.33)	0.62 (0.23)	0.33 (0.16)
Secondary+		Ref	Ref	Ref	Ref
TAF		0.84	0.85	0.70	0.44
p-value		0.000	0.000	0.000	0.006
**Income level** ^*^					
Low income		No data	0.33 (0.18)	0.57 (0.40)	0.71 (0.52)
Average income			0.16 (0.04)	0.57 (0.11)	0.64 (0.10)
High income			Ref	Ref	Ref
TAF			0.22	0.51	0.62
p-value			0.002	0.000	0.000
Parity*					
Nulliparous		Ref	Ref	Ref	Ref
Para 1-3		0.09 (0.04)	0.33 (0.15)	0.23 (0.10)	0.44 (0.21)
Para 4**+**		0.23 (0.08)	0.50 (0.19)	0.57 (0.23)	0.71 (0.26)
TAF		0.12	0.34	0.33	0.47
p-value		0.423	0.007	0.000	0.000
**Lacked skilled attendant at birth**					
	**1988**	**1993**	**1998**	**2003**	**2008**
	**AF (sTAF)**	**AF (sTAF)**	**AF (sTAF)**	**AF (sTAF)**	**AF (sTAF)**
**Residence** ^*^					
Urban	Ref	Ref	Ref	Ref	Ref
Rural	0.77 (0.66)	0.88 (0.80)	0.77 (0.70)	0.66 (0.59)	0.58 (0.50)
TAF	0.66	0.80	0.70	0.59	0.50
p-value	0.000	0.000	0.000	0.000	0.000
**Highest educational level** ^*^					
No education	0.80 (0.44)	0.85 (0.46)	0.85 (0.51)	0.84 (0.49)	0.73 (0.38)
Basic education	0.55 (0.23)	0.55 (0.24)	0.62 (0.24)	0.68 (0.26)	0.47 (0.22)
Secondary+	Ref	Ref	Ref	Ref	Ref
TAF	0.67	0.70	0.75	0.75	0.60
p-value	0.000	0.000	0.000	0.000	0.000
**Income level** ^*^		No data			
Low income	0.41 (0.20)		0.41 (0.20)	0.77 (0.57)	0.77 (0.59)
Average income	0.23 (0.04)		0.09 (0.02)	0.63 (0.11)	0.52 (0.07)
High income	Ref		Ref	Ref	Ref
TAF	0.24		0.22	0.68	0.66
p-value	0.000		0.001	0.000	0.000
Parity*					
Nulliparous	Ref	Ref	Ref	Ref	Ref
Para 1-3	0.38 (0.17)	0.44 (0.22)	0.47 (0.21)	0.44 (0.20)	0.44 (0.16)
Para 4+	0.38 (0.15)	0.50 (0.17)	0.71 (0.28)	0.62 (0.24)	0.62 (0.20)
TAF	0.32	0.39	0.49	0.44	0.36
p-value	0.007	0.001	0.000	0.000	0.001

*Mutually adjusted for one another, age, marital status, and the clustering at individual and household levels.

## Discussion

The key and most salient finding of this study is that the rural/urban gap, as well as educational inequalities in the utilization of ANC and SBA seem to be closing over time, while income inequality in the use of ANC is on a sharp rise. Income inequality regarding the utilization of SBA was rather low and stable from 1988 to 1998, increased sharply to a peak between 1998 and 2003, and then leveled-off after 2003. The increased utilization of ANC by women with low education and those who had rural residence status did not directly translate into utilization of SBA as would be expected. The utilization of SBA increased mainly in women with urban residence, high education, high income, and low parity. Despite the declines in inequalities observed in this study, there still remain significant negative associations between receiving the recommended number of ANC visits and SBA utilization, and rural residence status, no education, up to basic level education, low income, and high parity.

The declining trends in rural/urban gaps and education-related inequalities in ANC and SBA utilization is quite encouraging but what is most worrisome is that notwithstanding attempts by the government of Ghana to eliminate financial barriers to maternal health services utilization by vulnerable women, income inequalities in maternal health services utilization remain high. The results (Figures 1, 2 and 3) showed that, two decades ago, income-related inequalities in ANC and SBA utilization in Ghana were minimal, unlike in Kenya where income-related inequality in the use of SBA has always been high and persistent over the past two decades [42].

In Ghana, before 1998, low education and rural residence status almost solely contributed to non-use of ANC and SBA. From 1998 to 2003, the sharp increase in income-based inequality in the use of SBAs could be a reflection of the poor outcome of previous health care financing policies implemented in Ghana [43, 44]. In the early 1980s, Ghana implemented the cost recovery (popularly known as “cash and carry”) health care financing policy in all government facilities that required users to pay upfront before receiving care. This was meant to generate revenue and discourage ‘overuse’ of health services [43], but rather led to poor use of health services across the country. Moreover, income inequality has grown in Ghana since the late 1980s [45]. In 1987, income inequality, as measured by the Gini coefficient was 0.38, but it increased to 0.42 by 1998 and to 0.55 in 2007. Between 1992 and 1999, the mean income of the richest 10 percent of workers increased by 600 percent, while that of the poorest 10 percent increased by only 38 percent [45]. The high cost of health services coupled with the widening income inequalities in Ghana in the 1990s and early 2000 [45] is a plausible explanation for the sharp increase in income-related inequality in the utilization of maternal health services before year 2003, as seen in Figures 1, 2 and 3.

In 2005, exemptions for delivery care were introduced nationwide as an extension of the 2003 policy that was tested in four regions in Ghana. Later in 2008, free coverage was given to all pregnant women under the National Health Insurance Scheme, which begun in 2005 and overruled the previous exemption policy. Implementation of the exemptions policy could possibly be a factor for the stabilized income-related inequality seen in use of SBAs between 2003 and 2008. However, as shown in Table 4, within the same period, the situation for the average income women improved, whereas that of the very poor women worsened, though slightly. Also, income-related inequalities in the use of ANC continued to increase between 2003 and 2008. This implies that despite the potential of the delivery exemption policy to be pro-poor, they are in fact not reaching the poorest women [46], but paving the way for the average and high income women to increase their utilization of the services to the disadvantage of the poor women [47]. The exemption policy had aimed to remove financial barriers for accessing maternal health services and hence reduce maternal mortality [48]; however, implementation challenges have hindered the realization of this goal [47, 49, 50].

The free maternal health care policy lacks a clear source of funding [50, 51], relying on health care institutions to adjust for income inequalities in the use of maternal health services. The lack of funds means that pregnant women who access free maternal care put pressure on existing resources within health care institutions, forcing them to improvise other means of financing [52] by charging fees to women. This is done by charging for medications that are not on the National Health Insurance Scheme approved drug list, collecting ward fees and fees for toiletries. In some instances, women who seek care under the “free” maternal health care policy are discriminated against because they do not bring direct payments [44]. Although the plan is supposed to include all maternal and incidental causes, the package of care is interpreted differently by local health care institutions, resulting in pregnant women paying for services they ought to have received for free [47].

There are other factors on the provider side that tend to escalate the cost of maternal health services utilization for poor women, such as unavailability of critical equipment and drugs at the point of need, unavailability of skilled staff, and the poor attitude of providers [23, 47, 53], especially towards women who cannot afford the informal charges [21, 54]. Societal factors also drive informal payments and exacerbate the widening income-based inequalities in accessing maternal health care. One such factor is the culture of giving gifts to midwives, which come at a substantial cost to poor women [47]. Other indirect financial barriers include transport cost to and from the health facility [55], and minor personal items required for delivery [23, 47]. Some expectant mothers tend to be excessively delayed during antenatal visits, trading-off economic gains from the informal sector such as farming or petty trading [21, 53]. These factors have the potential to adversely affect poor women’s utilization of ANC and SBA, and also make it possible for high-income women to pay their way through the system, contributing to further widening the income inequality gap in the use of maternal health services in Ghana.

Most of the demands made upon women come at a time when they are about to give birth [21]. This is one of the reasons why the increased ANC uptake among low socio-economic status women did not translate into utilization of skilled care during delivery [47]. Education-related disparities in ANC utilization are decreasing and in SBA use remain large (Figures 2 and 3). Policy makers and program administrators need to review possible mechanisms, for example finding ways to improve timely transportation, removing any remaining financial bottlenecks, and providing education and/or additional incentives.

Thus, despite the huge potential for education to improve women’s utilization of maternal health services [56], too much direct and indirect cost excludes uptake of these services by some women who, although educated, lack financial resources [53]. Education improves a woman’s ability to evaluate where and when to seek care [57] and makes her aware of health services [58]. Higher education beyond the basic level empowers women to broaden their social network within the health care system, positions them to approach health care staff on more equal terms (making them less likely to fear possible reprimands), and makes them cognizant of private health facilities [59]. This may explain the educational gradient seen in Table 3.

Parity-related inequality in ANC utilization appears to be increasing over time, whereas that of SBA rose to a peak in 1998 and began to decline. This is due to reduced utilization of these services by multiparous women and increased uptake by nulliparous women. The use of ANC and SBA by expectant mothers may be influenced by their previous experience, either positively enhancing uptake, or negatively, discouraging use of services [53]. While multiparous women rely on their previous experiences [53], nulliparous hasten their use of antenatal and skilled delivery care [59], and even when they are faced with economic challenges, they mobilize external support to access care because of the value placed on first birth [59, 60]. Although some women may not utilize SBA, they may attend at least one ANC visit to obtain an antenatal card in case of eventual delivery at a health facility, being less concerned about monitoring the progress of the pregnancy [59]. This also provides a possible explanation to the almost universal single visit antenatal coverage. In other instances, women may continue antenatal visits to confirm that her pregnancy is progressing well and once reassured, may not seek skilled care at the time of delivery [61].

### Methodological considerations

One limitation of this study is that utilization of SBA was based on self reports. This could potentially have resulted in reporting bias due to some women’s inability to differentiate between a skilled birth attendant (doctor, nurse, or midwife) and one who is not. However, this potential bias was minimised by analysing each woman’s latest birth experience. Also, there was no data on the composition and quality of antenatal care received which could have given a more informed idea on the adequacy of care.

The declining trend in rural/urban gap in the use of maternal health services may partly be attributed to changes in infrastructure and improved acces to maternal health care services. The Community-based Health Planning and Services (CHPS) may be one such contributing factor [62–64]. This is a national health policy initiative adopted in 1999 that aims to reduce geographical barriers making access to health care difficult in Ghana by mainly focusing on deprived rural districts in remote areas. The CHPS strategy aims to transform the primary health care system in Ghana by establishing mobile community-based health care services provided by a resident nurse in deprived settings, contrary to traditional facility-based services [65]. However, the trend could also be explained by the potential bias in the way urban areas are defined in Ghana, mainly by population size [66]. Over the years, some rural areas have been reclassified as urban, although they still bear the unfavorable characteristics of rural areas, including poor infrastructure, inadequate transportation, low literacy levels, and low social status, among others. Also, the growing migration of people from rural areas into the cities has created a subset of the population in urban areas who live in slums where infrastructure and lifestyle factors are generally comparable to or worse than the places they left [67].

The level of participation in the surveys was generally very high. However, the 1988 survey lacked information on ANC. Thus our analysis of inequalities in ANC covered only 1993 to 2008. Also, there were high numbers of missing data on income in 1988 and 1998, and no income data in 1993. This could have potentially affected the income-related inequality estimations for 1988 and 1998. However, comparing the income distribution for 1988, 1998, 2003, and 2008 suggests that there is a fair representation of women across different income strata. Therefore, the overall effect of the missing data on income-related inequality estimates should be minimal. Another limitation related to income was the difficulty of measuring income levels for low-income women whose economic earnings are mostly from selling food or other items, or from small-scale farming. This has the potential to bias the results towards the null and underestimate the income inequalities observed.

Lastly, the commuting time/distance to health facility could be a potential confounder for not utilizing ANC and SBA that was not accounted for. However, this is unlikely to grossly affect the overall inequality estimation over time.

## Conclusions

The increased income-related inequalities seen in the use of antenatal care and skilled birth attendance should be addressed through appropriate strategies. Patient-provider communication training for health professionals both in health training schools and on the job should be re-enforced/strengthened. Intensifying community-based health education could further reduce education- and parity-related inequalities in skilled birth attendance and antenatal care utilization. The Ghana National Commission for Civic Education (NCCE) could create awareness on the use of maternal health services through media and door-to-door campaigns in deprived communities where women with no education could be reached. Women should be highly motivated and incentivized to attend school up to secondary level or higher. This could be achieved through interventions such as sustainable school feeding programs at the basic level and the introduction of tuition-free secondary level or higher education. Education on the use of maternal health services should also be intensified in basic schools so that women who end up at the basic level would have been inoculated with the appropriate health messages. This approach has two potentials: 1) to reach out to girls currently in basic schools and 2) indirectly reach out to their parents and other multiparous women in the society through diffusion of these health education messages to the local communities.

We recommend that information on the composition and quality of antenatal care received by women should be included in the standard DHS questionnaire so that future research can track inequalities in the standards of antenatal care received over time.

## References

[1] Zureick-Brown S, Newby H, Chou D, Mizoguchi N, Say L, Suzuki E, Wilmoth J (2013). Understanding global trends in maternal mortality. Int Perspect Sex Reprod Health.

[2] Mohammed AA, Elnour MH, Mohammed EE, Ahmed SA, Abdelfattah AI (2011). Maternal mortality in Kassala State - Eastern Sudan: community-based study using reproductive age mortality survey (RAMOS). BMC Pregnancy Childbirth.

[3] Aa I, Grove MA, Haugsja AH, Hinderaker SG (2011). High maternal mortality estimated by the sisterhood method in a rural area of Mali. BMC Pregnancy Childbirth.

[4] Dingle A, Powell-Jackson T, Goodman C (2013). A decade of improvements in equity of access to reproductive and maternal health services in Cambodia, 2000–2010. Int J Equity Health.

[5] Vallieres F, Cassidy EL, McAuliffe E, Isselmou SO, Hamahoullah MS, Lang J (2013). Where are the gaps in improving maternal and child health in Mauritania? the case for contextualised interventions: a cross sectional study. Pan Afr Med J.

[6] Joharifard S, Rulisa S, Niyonkuru F, Weinhold A, Sayinzoga F, Wilkinson J, Ostermann J, Thielman NM (2012). Prevalence and predictors of giving birth in health facilities in Bugesera District, Rwanda. BMC Public Health.

[7] Rahman M, Haque SE, Mostofa MG, Tarivonda L, Shuaib M (2011). Wealth inequality and utilization of reproductive health services in the Republic of Vanuatu: insights from the multiple indicator cluster survey, 2007. Int J Equity Health.

[8] Hussein J, Bell J, Nazzar A, Abbey M, Adjei S, Graham W (2004). The skilled attendance index: proposal for a new measure of skilled attendance at delivery. Reprod Health Matters.

[9] De Brouwere V, Tonglet R, Van Lerberghe W (1998). Strategies for reducing maternal mortality in developing countries: what can we learn from the history of the industrialized West?. Trop Med Int Health.

[10] Hogberg U, Wall S, Brostrom G (1986). The impact of early medical technology on maternal mortality in late 19th century Sweden. Int J Gynaecol Obstet.

[11] Fauveau V, Stewart K, Khan SA, Chakraborty J (1991). Effect on mortality of community-based maternity-care programme in rural Bangladesh. Lancet.

[12] WHO (2004). Making Pregnancy Safer: The Critical Role of the Skilld Attendant.

[13] Murakami I, Egami Y, Jimba M, Wakai S (2003). Training of skilled birth attendants in Bangladesh. Lancet.

[14] Harvey SA, Ayabaca P, Bucagu M, Djibrina S, Edson WN, Gbangbade S, McCaw-Binns A, Burkhalter BR (2004). Skilled birth attendant competence: an initial assessment in four countries, and implications for the Safe Motherhood movement. Int J Gynaecol Obstet.

[15] Ten Hoope-Bender P, Liljestrand J, MacDonagh S (2006). Human resources and access to maternal health care. Int J Gynaecol Obstet.

[16] Stanton C, Blanc AK, Croft T, Choi Y (2007). Skilled care at birth in the developing world: progress to date and strategies for expanding coverage. J Biosoc Sci.

[17] Kabakyenga JK, Ostergren PO, Turyakira E, Pettersson KO (2012). Influence of birth preparedness, decision-making on location of birth and assistance by skilled birth attendants among women in south-western Uganda. PLoS One.

[18] Holzgreve W, Greiner D, Schwidtal P (2012). Maternal mortality in Eritrea: Improvements associated with centralization of obstetric services. Int J Gynaecol Obstet.

[19] Koblinsky M, Matthews Z, Hussein J, Mavalankar D, Mridha MK, Anwar I, Achadi E, Adjei S, Padmanabhan P, Marchal B, De Brouwere V, van Lerberghe W (2006). Going to scale with professional skilled care. Lancet.

[20] Hounton S, Chapman G, Menten J, De Brouwere V, Ensor T, Sombie I, Meda N, Ronsmans C (2008). Accessibility and utilisation of delivery care within a Skilled Care Initiative in rural Burkina Faso. Trop Med Int Health.

[21] Bazzano AN, Kirkwood B, Tawiah-Agyemang C, Owusu-Agyei S, Adongo P (2008). Social costs of skilled attendance at birth in rural Ghana. Int J Gynaecol Obstet.

[22] Moyer CA, Adongo PB, Aborigo RA, Hodgson A, Engmann CM, Devries R (2014). “It’s up to the woman’s people”: how social factors influence facility-based delivery in Rural Northern Ghana. Matern Child Health J.

[23] Crissman HP, Engmann CE, Adanu RM, Nimako D, Crespo K, Moyer CA (2013). Shifting norms: pregnant women’s perspectives on skilled birth attendance and facility-based delivery in rural Ghana. Afr J Reprod Health.

[24] Wick L, Hassan S (2012). No safe place for childbirth: women and midwives bearing witness, Gaza 2008–09. Reprod Health Matters.

[25] Kabakyenga JK, Ostergren PO, Emmelin M, Kyomuhendo P, Odberg Pettersson K (2011). The pathway of obstructed labour as perceived by communities in south-western Uganda: a grounded theory study. Glob Health Action.

[26] Pettersson KO, Christensson K, de Freitas Eda G, Johansson E (2004). Adaptation of health care seeking behavior during childbirth: focus group discussions with women living in the suburban areas of Luanda, Angola. Health Care Women Int.

[27] Anwar I, Sami M, Akhtar N, Chowdhury ME, Salma U, Rahman M, Koblinsky M (2008). Inequity in maternal health-care services: evidence from home-based skilled-birth-attendant programmes in Bangladesh. Bull World Health Organ.

[28] Ochako R, Fotso JC, Ikamari L, Khasakhala A (2011). Utilization of maternal health services among young women in Kenya: insights from the Kenya Demographic and Health Survey, 2003. BMC Pregnancy Childbirth.

[29] WHO (2013). World Health Statistics 2013.

[30] Barros AJ, Ronsmans C, Axelson H, Loaiza E, Bertoldi AD, Franca GV, Bryce J, Boerma JT, Victora CG (2012). Equity in maternal, newborn, and child health interventions in Countdown to 2015: a retrospective review of survey data from 54 countries. Lancet.

[31] Villar J, Ba’aqeel H, Piaggio G, Lumbiganon P, Miguel Belizan J, Farnot U, Al-Mazrou Y, Carroli G, Pinol A, Donner A, Langer A, Nigenda G, Mugford M, Fox-Rushby J, Hutton G, Bergsjø P, Bakketeig L, Berendes H, Garcia J (2001). WHO antenatal care randomised trial for the evaluation of a new model of routine antenatal care. Lancet.

[32] Carroli G, Villar J, Piaggio G, Khan-Neelofur D, Gulmezoglu M, Mugford M, Lumbiganon P, Farnot U, Bersgjo P (2001). WHO systematic review of randomised controlled trials of routine antenatal care. Lancet.

[33] Boerma JT, Bryce J, Kinfu Y, Axelson H, Victora CG (2008). Mind the gap: equity and trends in coverage of maternal, newborn, and child health services in 54 Countdown countries. Lancet.

[34] Ghana Statistical Service, Ghana Health Service, and ICF Macro (2009). Ghana Demographic and Health Survey 2008: Key Findings.

[35] Nesbitt RC, Lohela TJ, Manu A, Vesel L, Okyere E, Edmond K, Owusu-Agyei S, Kirkwood BR, Gabrysch S (2014). Correction: quality along the continuum: a health facility assessment of intrapartum and postnatal care in Ghana. PLoS One.

[36] Nesbitt RC, Lohela TJ, Manu A, Vesel L, Okyere E, Edmond K, Owusu-Agyei S, Kirkwood BR, Gabrysch S (2013). Quality along the continuum: a health facility assessment of intrapartum and postnatal care in Ghana. PLoS One.

[37] Ghana Statistical Service (1989). Ghana Demographic and Health Survey 1988.

[38] Ghana Statistical Service (1994). Ghana Demographic and Health Survey 1993.

[39] Ghana Statistical Service, Macro International (1999). Ghana Demographic and Health Survey 1998.

[40] Ghana Statistical Service, Noguchi Memorial Institute for Medical Research N, ORC Macro (2004). Ghana Demographic and Health Survey 2003.

[41] Moussa KM, Ostergren PO, Eek F, Kunst AE (2010). Are time-trends of smoking among pregnant immigrant women in Sweden determined by cultural or socioeconomic factors?. BMC Public Health.

[42] Van Malderen C, Ogali I, Khasakhala A, Muchiri SN, Sparks C, Van Oyen H, Speybroeck N (2013). Decomposing Kenyan socio-economic inequalities in skilled birth attendance and measles immunization. Int J Equity Health.

[43] Barimah KB, Mensah J (2013). Ghana’s National Health Insurance Scheme: insights from members, administrators and health care providers. J Health Care Poor Underserved.

[44] Dalinjong AP, Laar AS (2012). The national health insurance scheme: perceptions and experiences of health care providers and clients in two districts of Ghana. Health Econ Rev.

[45] Obeng-Odoom F (2012). Neoliberalism and the Urban Economy in Ghana: Urban Employment, Inequality, and Poverty. Growth Change.

[46] Kanchebe Derbile E, van der Geest S (2013). Repackaging exemptions under National Health Insurance in Ghana: How can access to care for the poor be improved?. Health Policy Plan.

[47] Witter S, Garshong B, Ridde V (2013). An exploratory study of the policy process and early implementation of the free NHIS coverage for pregnant women in Ghana. Int J Equity Health.

[48] Ghana Ministry of Health (2008). Implementation Guidelines for Financing Free Delivery through NHIS.

[49] Witter S, Adjei S (2007). Start-stop funding, its causes and consequences: a case study of the delivery exemptions policy in Ghana. Int J Health Plann Manage.

[50] Witter S, Garshong B (2009). Something old or something new? Social health insurance in Ghana. BMC Int Health Hum Right.

[51] McPake B, Witter S, Ensor T, Fustukian S, Newlands D, Martineau T, Chirwa Y (2013). Removing financial barriers to access reproductive, maternal and newborn health services: the challenges and policy implications for human resources for health. Hum Resour Health.

[52] Agyepong IA, Nagai RA (2011). “We charge them; otherwise we cannot run the hospital” front line workers, clients and health financing policy implementation gaps in Ghana. Health Policy.

[53] Arthur E (2012). Wealth and antenatal care use: implications for maternal health care utilisation in Ghana. Health Econ Rev.

[54] Andersen HM (2004). “Villagers”: differential treatment in a Ghanaian hospital. Soc Sci Med.

[55] Masters SH, Burstein R, Amofah G, Abaogye P, Kumar S, Hanlon M (2013). Travel time to maternity care and its effect on utilization in rural Ghana: a multilevel analysis. Soc Sci Med.

[56] Ahmed S, Creanga AA, Gillespie DG, Tsui AO (2010). Economic status, education and empowerment: implications for maternal health service utilization in developing countries. PLoS One.

[57] Ensor T, Cooper S (2004). Overcoming barriers to health service access: influencing the demand side. Health Policy Plan.

[58] Titaley CR, Dibley MJ, Roberts CL (2010). Factors associated with underutilization of antenatal care services in Indonesia: results of Indonesia Demographic and Health Survey 2002/2003 and 2007. BMC Public Health.

[59] Pell C, Menaca A, Were F, Afrah NA, Chatio S, Manda-Taylor L, Hamel MJ, Hodgson A, Tagbor H, Kalilani L, Ouma P, Pool R (2013). Factors affecting antenatal care attendance: results from qualitative studies in Ghana, Kenya and Malawi. PLoS One.

[60] Singh PK, Singh L (2014). Examining Inter-Generational Differentials in Maternal Health Care Service Utilization: Insights from the Indian Demographic and Health Survey. J Biosoc Sci.

[61] Amooti-Kaguna B, Nuwaha F (2000). Factors influencing choice of delivery sites in Rakai district of Uganda. Soc Sci Med.

[62] Awoonor-Williams JK, Feinglass ES, Tobey R, Vaughan-Smith MN, Nyonator FK, Jones TC (2004). Bridging the gap between evidence-based innovation and national health-sector reform in Ghana. Stud Fam Plann.

[63] Naariyong S, Poudel KC, Rahman M, Yasuoka J, Otsuka K, Jimba M (2012). Quality of antenatal care services in the Birim North District of Ghana: contribution of the community-based health planning and services program. Matern Child Health J.

[64] Adongo PB, Tapsoba P, Phillips JF, Tabong PT, Stone A, Kuffour E, Esantsi SF, Akweongo P (2013). The role of community-based health planning and services strategy in involving males in the provision of family planning services: a qualitative study in Southern Ghana. Reprod Health.

[65] Nyonator FK, Awoonor-Williams JK, Phillips JF, Jones TC, Miller RA (2005). The Ghana community-based health planning and services initiative for scaling up service delivery innovation. Health Policy Plan.

[66] Owusu G (2005). Small towns in Ghana: Justifications for their promotion under Ghana’s decentralisation programme. Afr Stud Q.

[67] Stephens C, Akerman M, Avle S, Maia PB, Campanario P, Doe B, Tetteh D (1997). Urban equity and urban health: using existing data to understand inequalities in health and environment in Accra, Ghana and Sao Paulo, Brazil. Environ Urban.

[CR68] The pre-publication history for this paper can be accessed here: http://www.biomedcentral.com/1471-2393/14/295/prepub

